# Ferric-Chelate Reductase FRO3 Is Involved in Iron Homeostasis in Table Grape and Enhanced Plant Tolerance to Iron-Deficient Conditions

**DOI:** 10.3390/ijms26115172

**Published:** 2025-05-28

**Authors:** Jianping Wang, Chenxiao Wang, Yutong Cui, Matthew Shi, Meiling Tang, Zhizhong Song

**Affiliations:** 1 Yantai Academy of Agricultural Science of Shandong, No. 28 Fangchenggang Street, Yantai 264000, China; 2 College of Horticulture, Ludong University, No. 186 Hongqizhong Road, Yantai 264025, China; 3 Wolfson College, University of Cambridge, No. 6 Downing Street, Cambridge CB3 9BB, UK; 4 Department of Plant Science, University of Cambridge, No. 22 Mill Road, Cambridge CB2 3EA, UK

**Keywords:** table grape, iron transport and homeostasis, ferric-chelate reductase, Fe-deficient stress

## Abstract

In plants, ferric-chelate reductase (FRO) plays a critical role in mediating extracellular iron (Fe) reduction, a process essential for cellular Fe homeostasis and abiotic stress tolerance. However, the biological functions and regulatory mechanisms of FRO proteins in fruit crops remain poorly characterized. Here, six *VvFRO* genes were identified in the table grape cultivar ‘Yanhong’. Transcriptional analysis revealed that root expression of these genes was mainly induced under Fe deficiency, Fe depletion, NaCl stress, and PEG-induced drought stress, respectively, but remained unchanged by low temperature (4 °C) or heat treatment (45 °C). Among them, *VvFRO3* exhibited the highest constitutive expression, predominantly in leaves, and was significantly up-regulated under Fe deficiency, Fe depletion, or NaCl treatment. Functional complementation assays demonstrated that heterologous overexpression of *VvFRO3* in the *Arabidopsis thaliana fro2* knockout mutant rescued its growth retardation phenotype, particularly under Fe-deficient conditions. This study advances our understanding of Fe uptake, transport, and homeostasis mechanisms in perennial fruit crops.

## 1. Introduction

Iron (Fe) is an essential trace element in plant cells, functioning as a critical cofactor in iron–sulfur proteins and cytochromes. It participates in diverse metabolic pathways, including photosynthesis, respiration, phytohormone synthesis, amino acid and purine metabolism, and DNA repair [1,2,3,4]. Iron fertilization is closely associated with the growth, development, fruit quality formation, and yield of fruit trees. Iron deficiency in soil—particularly prevalent in calcareous soils—severely restricts plant growth and development, directly compromising crop productivity and quality [5,6,7,8].

In soil, the majority of iron exists as Fe^3+^, which is poorly bioavailable to plants [5,7,8]. Studies on the molecular mechanisms governing iron uptake, transport, and distribution have primarily focused on model plants, such as *Arabidopsis thaliana* and rice (*Oryza sativa*) [5,6,7,8,9,10,11,12], with limited attention given to perennial fruit crops. The optimal iron concentration for plant growth ranges from 10^−9^ to 10^−4^ mol/L. However, under typical soil pH conditions, dissolved Fe^2+^ and Fe^3+^ concentrations fall below 10^−15^ mol/L, insufficient to sustain robust plant development [6,8].

Romheld and Marschener [10] first delineated two distinct iron acquisition strategies in plants. Strategy I (employed by dicots and non-graminaceous monocots): Root plasma membrane-localized H^+^-ATPases secrete protons to acidify the rhizosphere, solubilizing Fe^3+^. Ferric-chelate reductase (FRO) subsequently reduces Fe^3+^ to Fe^2+^, which is absorbed via iron-regulated transporter (IRT) [6,8,9,10,11]. Strategy II (common in graminaceous monocots) relies on enzymatic synthesis and secretion of phytosiderophores (e.g., mugineic acids) that chelate Fe^3+^, enabling uptake via specialized iron transporter systems. Notably, rice utilizes both Strategy I and Strategy II for iron acquisition [6,8,9,10,11]. The MA-Fe³⁺ chelates are subsequently internalized through a phytosiderophore-dependent transport mechanism mediated by Yellow Stripe (YS) or Yellow Stripe-Like (YSL) transmembrane transporter systems [6].

Recent studies on the molecular mechanisms of iron uptake mediated by FROs has predominantly focused on annual model plants, such as *Arabidopsis thaliana* [10,12,13] and rice (*Oryza sativa*) [11]. The *AtFRO2* gene (AT1G01580) was first cloned from *Arabidopsis* roots, and complementation of the Fe reductase deficient mutant *fro2* with *AtFRO2* alleviated iron deficiency-induced growth inhibition and significantly enhanced FRO activity at the root surface in transgenic lines [12,13]. In rice, *OsFRO1* and *OsFRO7* are highly expressed in flag leaves, with their transcript levels regulated by abiotic stressors, such as NaCl, polyethylene glycol (PEG), high temperature, and heavy metals. RNAi knockdown lines of *OsFRO1* exhibited stunted growth, reduced iron and chlorophyll content, and diminished reactive oxygen species (ROS) scavenging capacity [11,14]. To date, FRO homologs have only been identified in *Citrus junos* cv. Ziyangxiangcheng [15], *Malus xiaojinensis* [16,17,18], and mango (*Mangifera indica*) [19], leaving the molecular basis of iron nutrition and utilization efficiency in perennial fruit trees poorly understood. In *M*. *xiaojinensis*, *MxFRO4* confers iron and salt tolerance through up-regulating antioxidant capacity associated with the ROS scavenging [17] and *MxFRO6* is implicated in iron and salt tolerance in *A. thaliana* [18].

Table grape (*Vitis vinifera*), a globally significant fruit crop, has a well-annotated genome [20]. Iron, as the highest amount among trace elements in grape trees, regulates grape quality and yield [2,5,7]. Nevertheless, the biological functions of FROs in grape remain uncharacterized. In this study, we identified and cloned FRO family genes from the independently bred cultivar ‘Yanhong’. Tissue-specific expression patterns were analyzed via quantitative real-time PCR (qRT-PCR), and the functionality of *VvFRO3* was validated through genetic complementation assays in the *Arabidopsis fro2* mutant. This study provides a theoretical foundation for elucidating iron uptake and transport mechanisms in perennial woody fruit trees.

## 2. Results

### 2.1. Identification and Isolation of VvFRO Family Genes in Table Grape

**Figure 1:**
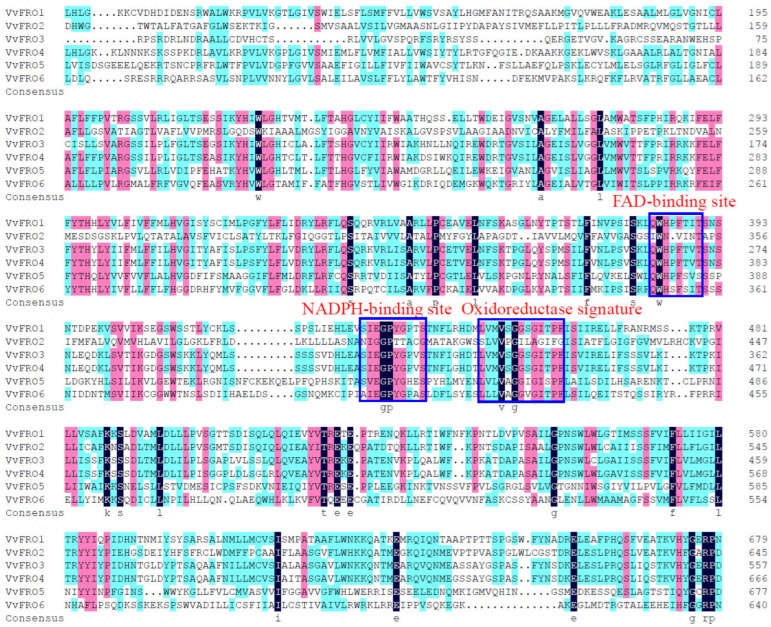
Amino acid sequence alignment of VvFRO proteins.

Using the amino acid sequences of *Arabidopsis* AtFROs as references [6,12], six putative FRO family proteins were identified through mining the grape genome database. The coding sequences (CDS) of these *FRO* genes were downloaded, and their full-length CDS were amplified from the ‘Yanhong’ table grape using high-fidelity DNA polymerase. After sequencing validation, the genes were designated *VvFRO1* to *VvFRO6* and CDS sequences were submitted to Genbank via National Center for Biotechnology Information (NCBI) website to obtain relevant numbers (Figure 1 and Table 1). All encoded proteins were predicted to contain functional domains critical for iron reductase activity, including the FAD-binding site, NADPH-binding site, and oxidoreductase signature motif (Figure 1), confirming their classification as canonical iron reductase family members.

**Figure 2:**
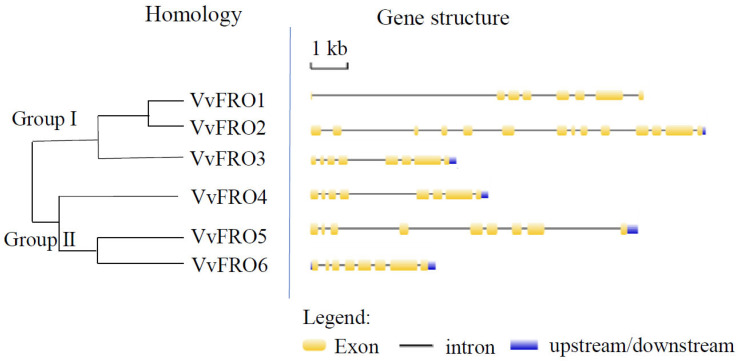
Homology and gene structure analysis of *VvFRO* genes.

Notably, *VvFRO* genes are predominantly located on chromosomes 12 (*VvFRO5*), 15 (*VvFRO3* and *VvFRO4*), 16 (*VvFRO1* and *VvFRO2*), and 17 (*VvFRO6*) (Table 1), all of which contain at least 7 introns of varying lengths (Figure 2). In addition, the amino acid sequence identity of VvFRO proteins is 51.62% (Figure 1) and the nucleotide sequence identity is 48.65%. Homology analysis indicated that VvFRO members can be divided into two Groups, and VvFRO1–VvFRO3 clustered within Group I, whereas VvFRO4–VvFRO6 were assigned to Group II (Figure 2).

**Figure 3:**
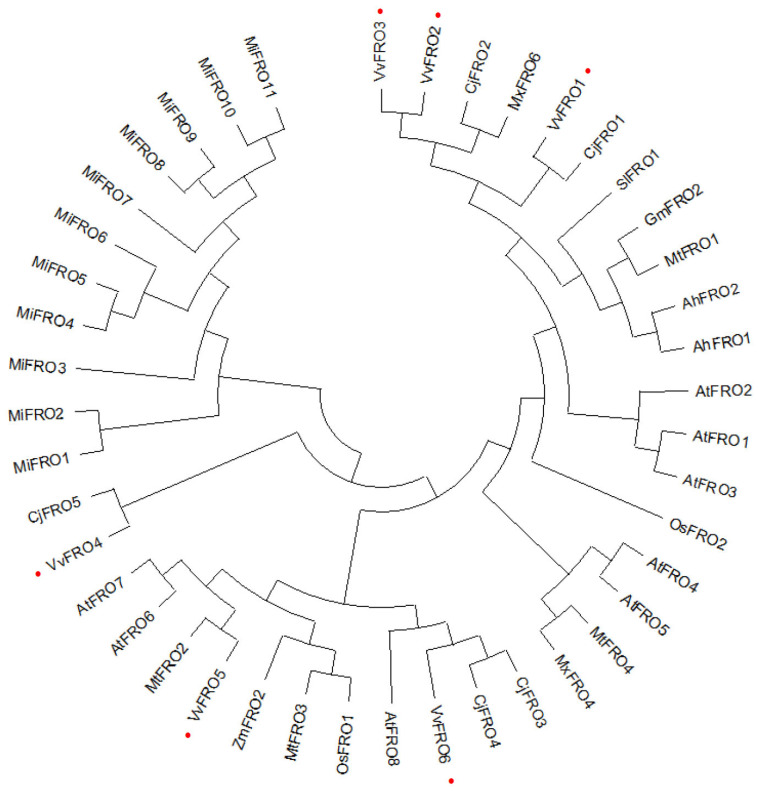
Phylogenetic tree of plant FRO homologs. A phylogenetic tree was constructed using the Maximum Likelihood method in MEGA 13.0. Table grape VvFRO proteins are labelled with red dot.

Comparative analysis of FRO proteins from 11 plant species across distinct families revealed high homology, with an amino acid sequence identity of 43.66%, and closely related homologs displayed sequence identities exceeding 67.35%. Phylogenetic tree analysis indicated that evolutionary relationships among FRO proteins from 10 plant species showed distinct clustering patterns. In particular, VvFROs exhibited closer phylogenetic proximity to homologs from *C. junos* CjFROs and *M. xiaojinensis* MxFROs (Figure 3). For instance, VvFRO1 and VvFRO4 clustered tightly with CjFRO1 and CjFRO5, respectively, while VvFRO6 grouped closely with CjFRO3 and CjFRO4. Similarly, VvFRO2 and VvFRO3 showed strong clustering with MxFRO6. In contrast, FRO homologs from legume species—soybean GmFRO2, barrel medic MtFRO1, and peanut AhFRO1, AhFRO2)—formed a distinct clade, reflecting their shared taxonomic lineage. Similarly, homologs from monocot species, rice OsFRO1 and maize ZmFRO2, exhibited the closest genetic distance (Figure 3).

### 2.2. Expression Profiles of VvFRO Genes

**Figure 4:**
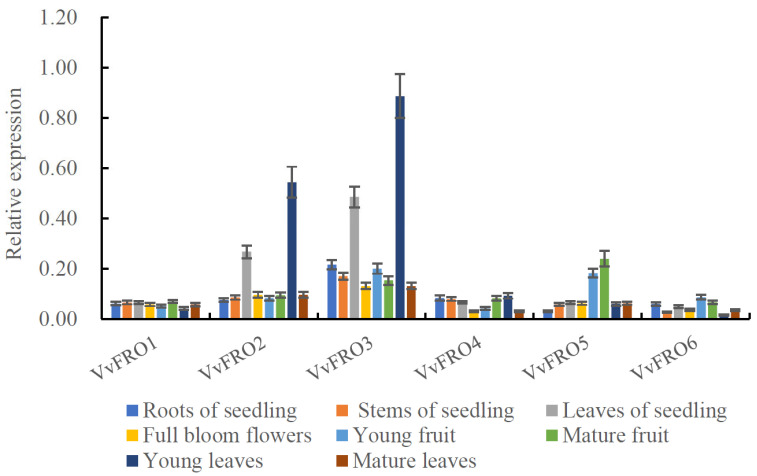
Tissue specific expression analysis of VvFRO *genes*.

qRT-PCR analysis revealed differential expression levels of VvFRO1*–VvFRO6* in various tissues of ‘Yanhong’ tissue-cultured seedlings and adult trees (Figure 4). *VvFRO3* exhibited the highest overall expression across tissues, and the highest levels were observed in young leaves of adult trees and seedling leaves, higher than that of the other tested tissues (Figure 4). *VvFRO2* showed a similar expression profile to *VvFRO3*. Moreover, *VvFRO5* displayed higher expression in fruits (either young or mature fruit) compared to other tissues. The remaining three genes (*VvFRO1*, *VvFRO4*, and *VvFRO6*) exhibited relatively low expression levels (all < 0.1 and values are closely comparable) across different table grape tissues (Figure 4).

**Figure 5:**
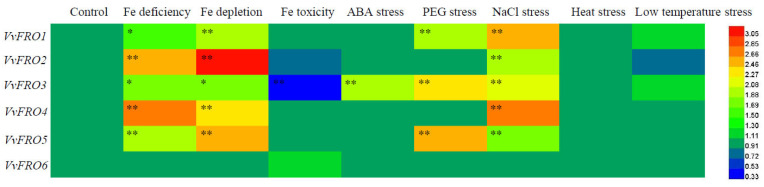
Expression analysis of *VvFRO* genes under different abiotic stresses. One-month-old tissue cultured seedlings were subjected to Fe deficiency (50% Fe was removed from the MS solution), Fe depletion (100% Fe was removed from the MS solution), Fe toxicity (500 μmol∙L^−1^ FeCl_3_), ABA (200 µmol∙L^−1^ ABA), drought (10% PEG6000, *w*/*v*), NaCl (150 mmol∙L^−1^ NaCl), low temperature (4 °C), high temperature (4 °C) treatment for 48 h before q-RT-PCR analysis. Asterisks indicate statistical differences found between the control and abiotic stress treatment using Student’s *t*-test in SPSS 13.0 software (* *p* < 0.05, ** *p* < 0.01).

At the transcriptional level, the responses of *VvFRO1*–*VvFRO6* in the roots of ‘Yanhong’ seedlings to eight different abiotic stresses exhibited distinct variations (Figure 5). The *VvFRO* family genes were most sensitive to Fe deficiency, Fe depletion, and NaCl stress. With the exception of *VvFRO6*, the expression levels of the other five *VvFRO* genes in roots were significantly induced under Fe deficiency, Fe depletion, and NaCl-treated conditions. Three genes (*VvFRO1*, *VvFRO3*, and *VvFRO5*) showed marked sensitivity to PEG treatment, with their root expression levels significantly up-regulated. Notably, only *VvFRO3* responded to abscisic acid (ABA) treatment, displaying a significant induction in root expression, and it was also the sole gene suppressed under Fe toxicity, with its expression significantly down-regulated. In contrast, the *VvFRO* family genes were insensitive to temperature fluctuations, as no significant changes in expression were observed under either low-temperature (4 °C) or heat (45 °C) stress.

Specifically, three genes (*VvFRO1*, *VvFRO3*, and *VvFRO5*) exhibited transcriptional plasticity in roots, responding to at least three stress treatments. Among these, *VvFRO3* displayed the highest overall expression levels in grape roots and responded to five stress conditions (excluding temperature extremes). Although *VvFRO6* exhibited relatively low basal expression, its transcriptional stability was striking, showing no response to any of the seven stress treatments tested in this study.

### 2.3. VvFRO3 Rescued the Impaired Growth of Arabidopsis fro2 Mutant

**Figure 6:**
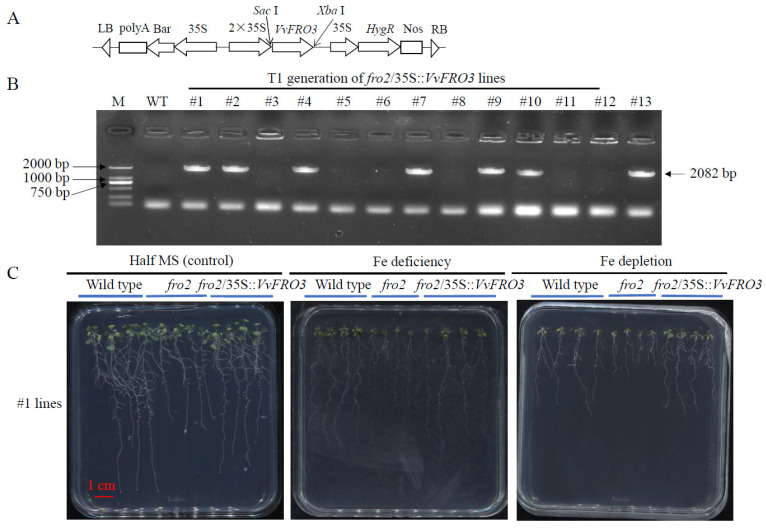
Generation and phenotype analysis of *VvFRO3* complementation *Arabidopsis* seedlings. (**A**) Construction of recombinant plasmid pBH-*VvFRO3*. (**B**) PCR verification of *VvFRO3* in T1 generation *fro2*/35S::*VvFRO3* lines. Note: M, standard DL2000 DNA ladder (Takara, Dalian, China). WT, wild type. (**C**) Phenotype analysis of T3 generation *fro2*/35S::*VvFRO3* lines. *Arabidopsis* seedlings were germinated on half-strength MS solid medium and subjected to Fe deficiency (50% Fe was removed from the MS solution) or Fe depletion (100% Fe was removed from the MS solution) for 7 days before phenotype analysis. Data are presented as means ± SE (*n* = 20). Bar = 1 cm.

In *Arabidopsis*, the growth of *fro2* knockout mutant was severely impaired [9,14]. To determine whether *VvFRO3* could restore the normal growth of *fro2* mutant, *VvFRO3* was introduced into the binary expression vector pHB (Figure 6A). At least seven putative (#1, #2, #4, #7, #9, #10, and #13) T1 generation *fro2*/35S::*VvFRO3* lines were validated using reverse transcription PCR for the presence of a 2082 bp fragment of *VvFRO3* (Figure 6B). Purified T3 generation of #1 and #13 *fro2*/35S::*VvFRO3* lines were randomly selected for further physiological analysis. Given that #1 and #13 *fro2*/35S::*VvFRO3* lines exhibited similar growth status, data of #1 *fro2*/35S::*VvFRO3* lines are shown in this present work.

Compared to the wild type, the growth of *fro2* lines was seriously hindered, accompanied by decreased fresh weight, dry weight, primary root length, lateral root numbers, and total leaf chlorophyll, under the control conditions, Fe deficiency, and Fe depletion, respectively (Figure 6C, Table 2). In contrast, #1 *fro2*/35S::*VvFRO3* lines exhibited a better growth status than that of the *fro2* mutant lines under all tested conditions. The fresh weight, dry weight, primary root length, lateral root numbers, and total leaf chlorophyll of #1 *fro2*/35S::*VvFRO3* lines were significantly increased, compared to the *fro2* mutant, similar to that of the wild type (Figure 6C, Table 2). These findings indicate that the complementation of *VvFRO3* rescued the impaired growth of *Arobidopsis fro2* mutants.

In comparison to the wild type, the tissue Fe content, ACO activity, NiR activity, and SDH activity of *fro2* mutant lines were significantly decreased under control conditions, Fe deficiency and Fe depletion, respectively (Table 2). Compared to the *fro2* mutant, the tissue Fe concentration, ACO activity, NiR activity, and SDH activity of #1 *fro2*/35S::*VvFRO3* lines were significantly induced (Table 2).

## 3. Discussion

Fe plays a critical role in fruit quality development and is closely associated with crop yield. However, the biological functions of genes involved in Fe uptake and translocation in fruit trees remain poorly characterized. As a Strategy I dicot species, grapevine absorbs Fe via rhizosphere acidification and FRO activity [5,6,8,9,10,11], yet the molecular mechanisms underlying Fe acquisition and transport remain largely unexplored. In this study, six *FRO* family genes were cloned and characterized from table grape (Figure 1). This number is slightly lower than homologs in *Arabidopsis* (8 *FRO* genes) [6,12] and mango (11 *FRO* genes) [19], suggesting lineage-specific divergence in *FRO* gene family size across plant taxa. Phylogenetic analysis further revealed distinct evolutionary relationships among FRO homologs from different plant families. Notably, VvFRO proteins clustered more closely with homologs from perennial woody species (*C. junos* and *M. xiaojinensis*) than with those from Poaceae, Fabaceae, Solanaceae, or Brassicaceae species (Figure 3), implying conserved biological roles of FRO enzymes in woody fruit crops. These findings suggest that, while FRO family members share close phylogenetic relationships, they may have undergone functional specialization during long-term evolution, highlighting adaptive evolution in plant Fe homeostasis mechanisms.

Previous studies have demonstrated high expression levels of *FRO* family genes in leaves, including *OsFRO1* and *OsFRO7* in rice [11], *CjFRO1* in *C. junos* [15], *MxFRO4* and *MxFRO6* in *M. xiaojinensis* [16,17,18], and *MiFRO4*, *MiFR8*, and *MiFR11* in mango [19]. Consistent with these findings, *VvFRO2* and *VvFRO3* exhibited elevated expression (2- to 4-fold higher than other tissues) in young leaves of mature grapevines and leaves of tissue-cultured seedlings (Figure 4), further supporting the critical role of *FRO* genes in leaf iron absorption and translocation. Notably, while no prior studies have reported *FRO* expression profiles in fruits, we observed relatively high expression of *VvFRO5* in fruits, with levels significantly exceeding those in leaves, roots, and other tissues, suggesting its potential involvement in Fe homeostasis during pivotal developmental stages of fruit maturation.

Previous studies have demonstrated that *OsFRO1* and *OsFRO7* in rice exhibit transcriptional responses to abiotic stressors, such as salt, drought, heat, and heavy metals [11], while *MxFRO4* and *MxFRO6* in *M. xiaojinensis* are modulated under NaCl stress [16,17,18]. Notably, *OsFRO7* is transcriptionally induced by ABA treatment, suggesting its potential role in osmotic regulation in rice, whereas *OsFRO1* lacks transcriptional responsiveness to ABA [11]. In contrast, our study revealed that only *VvFRO3* in grapevine roots exhibited significant upregulation under ABA treatment, implying its involvement in osmotic adjustment and providing a foundation for further exploration of FRO enzyme functions and regulatory networks in grapevine. In addition, *VvFRO1*, *VvFRO3*, and *VvFRO5* were transcriptionally induced in grapevine roots under PEG-mediated drought stress (Figure 5), suggesting potential roles in drought response pathways. Strikingly, despite grapevine’s limited adaptability to extreme climates, *VvFRO* genes exhibited no transcriptional changes under low-temperature (4 °C) or high-temperature (45 °C) treatment highlights. This expression stability may ensure the maintenance of essential iron metabolism and physiological processes, allowing grapevine to endure suboptimal thermal conditions. However, functional validation through targeted experiments is required to confirm these observations.

In the present study, the transcriptional regulation of *VvFRO* genes in table grape is responsive to Fe availability, with pronounced sensitivity to Fe deficiency and Fe depletion stresses. Specifically, five out of six *VvFRO* genes (excluding *VvFRO6*) exhibited significant up-regulation in roots under Fe-deficient conditions (Figure 5), suggesting that grape FRO family members are preferentially induced to maximize ferric-chelate reductase activity, thereby sustaining iron uptake, translocation, and Fe-dependent physiological processes in roots. The elevated expression of *FRO* genes is likely to serve as a critical signaling mechanism for grapevine adaptation to Fe-limited environments. Notably, *VvFRO3* displayed the highest constitutive expression across tissues in ‘Yanhong’, with root expression levels 3- to 7-fold higher than other *VvFRO* genes. Its expression was markedly suppressed under Fe toxicity and induced under Fe-deficient stresses, implying that *VvFRO3* encodes a highly active ferric-chelate reductase whose activity is directly modulated by external iron availability. These findings align with reported Fe-responsive regulation of FRO genes in *Arabidopsis* [12], *C. junos* [15], *M. xiaojinensis* [16,17,18], and mango [17]. In *M. xiaojinensis*, *MxFRO4* enhances iron and salt stress tolerance by upregulating antioxidant systems that scavenge reactive oxygen species (ROS) [17]. Complementary studies demonstrate that heterologous expression of *MxFRO6* in *Arabidopsis* similarly improves iron acquisition and salt tolerance in transgenic seedlings [18]. In this present study, heterologous expression of *VvFRO3* in *Arabidopsis fro2* mutant favorably rescued the retarded growth of *fro2* mutants. In addition, tissue Fe content and activity of Fe-dependent enzymes (ACO, NiR, and SDH) were significantly enhanced in *fro2*/35S::*VvFRO3* lines, which may partially account for the restored growth performance. Over-expression of *VvFRO3* in *fro2* mutant may positively strengthen the Fe uptake and transport capacity in *fro2*/35S::*VvFRO3* lines, maintaining basic Fe-dependent metabolic processes, thereby preventing the transgenic seedlings from Fe-deficient stresses. Meanwhile, tissue Fe content and total leaf chlorophyll were indeed induced in *fro2*/35S::*VvFRO3* lines. 

To date, numerous transcription factors and Fe uptake and transport-related functional genes have been associated with iron uptake and transport mechanisms, and such regulatory mechanisms have been documented in *Arabidopsis* systems [4,6]. Our previous work utilized *Arabidopsis fro2* mutants to functionally validate *VvFRO3* through physiological phenotyping. Future investigations will employ these genetic resources to conduct integrated proteomic and transcriptomic profiling under Fe deprivation conditions to identify transcription factors coordinating with VvFRO3-mediated Fe homeostasis and characterize post-translational modifications influencing VvFRO3 function. Nonetheless, this study favors the proposition that ferric-chelate reductase FRO3 is implicated in modulating Fe transport and homeostasis and plant adaptation to undesired Fe-deficient stresses.

## 4. Materials and Methods

### 4.1. Plant Material and Growth Condition

The new variety of table grape ‘Yanhong’ cultivated independently was used throughout this study. One-month-old tissue-cultured seedlings and 5-year-old mature ‘Yanhong’ trees were grown in the National Grape Germplasm Repository (Yantai, China).

For the control treatments, 1-month-old tissue-cultured seedlings were grown in half-strength Murashige and Skoog (MS) liquid solution. For Fe deficiency treatments, 50% Fe was removed from the MS solution. For Fe depletion treatments, 100% Fe was deleted from the MS solution [4,19,21]. For Fe toxicity treatments, 500 μmol∙L−1 FeCl3 was added in half-strength MS solution [19,22]. For ABA treatments, 200 µmol∙L−1 ABA was supplied in half-strength MS medium [17,21]. For PEG-mediated drought stress treatments, 10% PEG6000 (w/v) was added in half-strength MS solution [19,22,23,24]. For salt stress treatments, 150 mmol∙L−1 NaCl was added in half-strength MS solution [19,22,23,24]. For low temperature treatments, seedlings were placed in the 4 °C incubator [4]. For high temperature treatments, seedlings were placed in the 45 °C incubator [19]. After being subjected to stress treatment for 48 h, root samples were collected and frozen in liquid nitrogen before further analyses.

The wild type *Arabidopsis* (Col-0), *fro2* knockout mutants (purchased and verified from The Arabidopsis Information Resource, https://www.arabidopsis.org) [12,13], and *fro2*/35S::*VvFRO2* lines were germinated in half-strength MS medium and exposed to the control, Fe deficiency, or Fe depletion treatment for 7 days before physiological analysis. Three independent biological repeats were conducted, each comprising 20 *Arabidopsis* seedlings.

### 4.2. Physiological Analysis

Fresh weight of stress-treated Arabidopsis seedlings was measured using an Analytical Balance (Thermo Electron, Waltham, MA, USA). Primary root length and lateral root number were manually quantified by direct visual inspection [4,19,21,22,23]. Enzyme activities of aconitase (ACO), nitrite reductase (NiR), and succinate dehydrogenase (SDH) were assayed using commercially available detection kits (Nanjing Jiancheng Bioengineering Institute, Nanjing, China). Total leaf chlorophyll content was quantified with a BioRad SmartSpec 3000 spectrophotometer (Wadsworth, IL, USA) following previously established protocol [21,22,23]. Tissue Fe content was determined by ICP–AES systems (IRIS Advantage, Thermo Electron, Waltham, MA, USA). Three independent biological replicates were executed, each comprising 20 seedlings.

### 4.3. Isolation and Cloning of VvFRO Family Genes from Table Grape

Putative grape FRO family genes (VvFROs) were identified by interrogating the Phytozome grape genome database (http://www.phytozome.net) using amino acid sequences of eight Arabidopsis AtFRO proteins as queries [6,12]. Functional domains of VvFRO proteins were confirmed through analysis on the Pfam online server (Accessed on 26 March 2023, http://pfam.xfam.org/search) [19,21,23]. Gene-specific primers (Table 1) were designed based on the coding sequences (CDS) of VvFRO genes. PCR amplification was performed with the PrimeSTAR^TM^ HS DNA Polymerase Kit (TaKaRa, Dalian, China) for high-fidelity amplification, and products were validated by sequencing at Sangon Biotech Co., Ltd. (Shanghai, China).

### 4.4. Phylogenetic Tree Analysis

Following the description of Gao et al. [23] and Zhang et al. [24], amino acid sequences of FRO homologous proteins were retrieved from 11 plant species: grape (VvFRO, Rosaceae), *M. xiaojinensis* (MxFRO, Rosaceae), *C. junos* (CjFRO, Rutaceae), mango (MiFRO*,* Anacardiaceae), *Arabidopsis* (AtFRO, Brassicaceae), tomato (SlFRO, *Solanum lycopersicum*, Solanaceae), maize (ZmFRO, *Zea mays*, Poaceae), rice (OsFRO, *Poaceae*), barrel medic (MtFRO, *Medicago truncatula*, Fabaceae), peanut (AhFRO, *Arachis hypogaea*, Fabaceae), and soybean (GmFRO, *Glycine max*, Fabaceae). Multiple sequence alignment was performed using ClustalX 2.0 to analyze amino acid sequence conservation. A phylogenetic tree of plant FRO homologs was constructed with the Maximum Likelihood method in MEGA 13.0 to elucidate the genetic evolutionary relationships among FRO proteins across these species [23,24].

### 4.5. Quantitative Real Time PCR (qRT-PCR)

Gene-specific primer pairs of *VvFRO* genes were designed using the NCBI/Primer-BLAST on-line server and are provided in Table 3. qRT-PCR analysis was conducted on the 7500 Real Time PCR System (Applied Biosystems, Foster City, CA, USA) with SYBR Premix Ex Taq (TaKaRa, Dalian, China). The Ubiquitin of wine grape was employed as the internal control [22,25,26]. The relative expression levels of *VvFRO* genes were calculated via normalization to *Ubiquitin* using data from three independent biological replicates.

### 4.6. Overexpression of VvFRO3 in Arabidopsis fro2 Mutant

The CDS of VvFRO3 was cloned into the pBH vector [4,21,24] using the forward primer 5′-GACGAGCTCATGAGACCGCTTCTCTTGGTG-3′ (*Sac* I site underlined) and the reverse primer 5′ GAGTCTAGATCACCAGCTGAAGCTTATGGA-3′ (*Xba* I site underlined), yielding the recombinant plasmid pBH-*VvFRO3*. Both the empty pBH vector and pBH-VvFRO3 were transformed into Agrobacterium tumefa-ciens strain EHA 105 and subsequently introduced into Arabidopsis fro2 knockout homo-zygote mutants [9,13]. Transgenic T1 generation *fro2*/35S::*VvFRO3* lines were screened on hygromycin-supplemented solid medium. Genomic DNA was extracted from T1 *fro2*/35S::*VvFRO3* lines using the Universal Genomic DNA Extraction Kit (TaKaRa, Dalian, China), followed by PCR verification of a 2082 bp *VvFRO3* fragment. Validated T3 seeds of *fro2*/35S::*VvFRO3* lines were surface-sterilized and germinated on half-strength MS solid medium under controlled conditions for 7 days. Three biological replicates were conducted, each consisting of 20 seedlings.

### 4.7. Statistical Analysis

Statistical graphs were generated using OriginPro 12.0 software (OriginLab Corporation, Northampton, MA, USA). Significant differences were analyzed using Student’s *t*-test in SPSS 13.0 software (SPSS, Chicago, IL, USA) or ANOVA Fisher’s LSD test [4,19,21,23]. Please see details in the figure or table legends.

## 5. Conclusions

Six *VvFRO* genes were isolated from table grape ‘Yanhong’. *VvFRO3* was the most abundantly expressed gene, which was mainly expressed in leaves and was up-regulated under Fe deficiency, Fe depletion, or NaCl treatment. Over-expressing of *VvFRO3* rescued the retarded growth of *Arabidopsis fro2* knockout mutant, especially under Fe deficiency and Fe depletion. *VvFRO3* may be a crucial ferric-chelate reductase that is involved in Fe transport and homeostasis in table grape, especially under Fe-deficient conditions.

## Figures and Tables

**Table 1:** Information for *VvFRO* family genes.

Gene	Genbank No.	Accession ID	Chromosome Location	CDS (bp)	Intron
*VvFRO1*	PQ893568	GSVIVT01028874001	chr16:17911251..17919834 reverse	2184	8
*VvFRO2*	PQ893569	GSVIVT01028873001	chr16:17920090..17930273 reverse	3537	13
*VvFRO3*	PQ893570	GSVIVT01026993001	chr15:18731174..18734997 reverse	2082	7
*VvFRO4*	PQ893571	GSVIVT01026991001	chr15:18754824..18759433 reverse	2145	7
*VvFRO5*	PQ893572	GSVIVT01023105001	chr12:22531017..22539454 forward	2208	8
*VvFRO6*	PQ893573	GSVIVT01007662001	chr17:10745402..10748530 forward	2112	7

**Table 2:** Physiological analysis of *VvFRO3* complementation *Arabidopsis* seedlings.

Items	Treatment	Wild Type	*fro2* Mutant	#1 *fro2*/35S::*VvFRO3*
Fresh weight (g)	Control	2.63 ± 0.27 ^a(a)^	1.22 ± 0.13 ^a(b)^	2.53 ± 0.32 ^a(a)^
	Fe deficiency	0.81 ± 0.06 ^b(a)^	0.38 ± 0.03 ^b(b)^	0.76 ± 0.08 ^b(a)^
	Fe depletion	0.44 ± 0.03 ^c(a)^	0.20 ± 0.01 ^c(b)^	0.37 ± 0.04 ^c(a)^
Dry weight (g)	Control	0.22 ± 0.02 ^a(a)^	0.11 ± 0.01 ^a(b)^	0.21 ± 0.02 ^a(a)^
	Fe deficiency	0.07 ± 0.007 ^b(a)^	0.02 ± 0.003 ^b(b)^	0.06 ± 0.007 ^b(a)^
	Fe depletion	0.03 ± 0.004 ^c(a)^	0.01 ± 0.001 ^bc(b)^	0.03 ± 0.02 ^c(a)^
Primary root length (cm)	Control	10.54 ± 1.15 ^a(a)^	5.78 ± 0.61 ^a(b)^	10.13 ± 1.22 ^a(a)^
	Fe deficiency	6.69 ± 0.54 ^b(a)^	4.25 ± 0.52 ^b(b)^	6.64 ± 0.56 ^b(a)^
	Fe depletion	4.30 ± 0.41 ^c(a)^	2.68 ± 0.34 ^c(b)^	4.32 ± 0.37 ^c(a)^
Lateral root numbers	Control	24 ± 4 ^a(a)^	15 ± 3 ^a(b)^	22 ± 3 ^a(a)^
	Fe deficiency	17 ± 3 ^b(a)^	8 ± 1 ^b(b)^	17 ± 2 ^b(b)^
	Fe depletion	9 ± 2 ^c(a)^	4 ± 2 ^c(b)^	11 ± 2 ^c(a)^
Leaf total chlorophyll (g·kg^−1^ FW)	Control	1.67 ± 0.19 ^a(a)^	1.02 ± 0.022 ^a(b)^	1.56 ± 0.023 ^a(a)^
	Fe deficiency	0.53 ± 0.52 ^b(a)^	0.38 ± 0.045 ^b(b)^	0.57 ± 0.061 ^b(a)^
	Fe depletion	0.40 ± 0.043 ^bc(a)^	0.28 ± 0.034 ^bc(b)^	0.39 ± 0.046 ^c(a)^
ACO activity [U·(mg protein)^−1^]	Control	0.89 ± 0.091 ^a(a)^	0.53 ± 0.055 ^a(b)^	0.91 ± 0.098 ^a(a)^
	Fe deficiency	0.58 ± 0.063 ^b(a)^	0.32 ± 0.027 ^b(b)^	0.57 ± 0.062 ^b(a)^
	Fe depletion	0.39 ± 0.028 ^bc(a)^	0.21 ± 0.022 ^c(b)^	0.42 ± 0.031 ^bc(a)^
NiR activity [U·(mg protein)^−1^]	Control	1.48 ± 0.16 ^a(a)^	0.82 ± 0.095 ^a(b)^	1.39 ± 0.15 ^a(a)^
	Fe deficiency	0.83 ± 0.092 ^b(a)^	0.48 ± 0.051 ^b(b)^	0.87 ± 0.094 ^b(a)^
	Fe depletion	0.62 ± 0.073 ^bc(a)^	0.36 ± 0.025 ^bc(b)^	0.64 ± 0.059 ^bc(a)^
SDH activity [U·(mg protein)^−1^]	Control	21.45 ± 2.26 ^a(a)^	13.72 ± 1.67 ^a(b)^	20.69 ± 3.04 ^a(a)^
	Fe deficiency	15.77 ± 1.62 ^b(a)^	9.83 ± 0.87 ^b(b)^	15.36 ± 1.49 ^b(a)^
	Fe depletion	10.68 ± 1.47 ^c(a)^	6.79 ± 0.85 ^c(b)^	9.99 ± 1.23 ^c(a)^
Fe content (g·kg^−1^ DW)	Control	13.33 ± 1.45 ^a(a)^	7.89 ± 0.65 ^a(b)^	12.03 ± 1.51 ^a(a)^
	Fe deficiency	9.62 ± 0.91 ^b(a)^	5.01 ± 0.63 ^b(b)^	8.98 ± 0.91 ^b(a)^
	Fe depletion	4.77 ± 0.42 ^c(a)^	2.62 ± 0.02 ^c(b)^	4.58 ± 0.05 ^c(a)^

Note: *Arabidopsis* seedlings were germinated on half-strength MS solid medium and subjected to Fe deficiency (50% Fe was removed from the MS solution) or Fe depletion (100% Fe was removed from the MS solution) for 7 days before phenotype analysis. Data are presented as means ± SE (n = 20). Different letters outside the parentheses indicate differences among control condition, Fe deficiency, and Fe depletion, and those inside the parentheses indicate differences among wild type, *fro2* mutant, and *fro2*/35S::*VvFRO3* lines, respectively.

**Table 3:** Specific primer pairs used for qRT-PCR.

Gene	Forward (5′ → 3′)	Reverse (5′ → 3′)
*VvFRO1*	TGGAAGCAAAGGAAGCTCTTA	GAGGAGGTTGGCACCTTGTT
*VvFRO2*	GCCGTCATCCATCTCCCATC	CTCTGACCAGAGGCCGAAAG
*VvFRO3*	GCTCCTTCAACTCCGAGCAA	GGGCTGCTTATAGACTTATTATC
*VvFRO4*	TGCTTGCCTTGATGATCTTCCT	GCCGATCTTTTGGGCTGCTTT
*VvFRO5*	CCTTTTGGAGTGGTCTCTGCT	GGCGGAGAAGAACTGATCCC
*VvFRO6*	CCAAATCATTGAAGCGGCAGT	ATCATTGCAGTCCCAAGCCA

## Data Availability

The original contributions presented in the study are included in the article, further inquiries can be directed to the corresponding author.
